# Stimulus Specificity of Brain-Computer Interfaces Based on Code Modulation Visual Evoked Potentials

**DOI:** 10.1371/journal.pone.0156416

**Published:** 2016-05-31

**Authors:** Qingguo Wei, Siwei Feng, Zongwu Lu

**Affiliations:** Dept. of Electronic Engineering, School of Information Engineering, Nanchang University, Nanchang, 330029, China; Shanghai Jiao Tong University, CHINA

## Abstract

A brain-computer interface (BCI) based on code modulated visual evoked potentials (c-VEP) is among the fastest BCIs that have ever been reported, but it has not yet been given a thorough study. In this study, a pseudorandom binary M sequence and its time lag sequences are utilized for modulation of different stimuli and template matching is adopted as the method for target recognition. Five experiments were devised to investigate the effect of stimulus specificity on target recognition and we made an effort to find the optimal stimulus parameters for size, color and proximity of the stimuli, length of modulation sequence and its lag between two adjacent stimuli. By changing the values of these parameters and measuring classification accuracy of the c-VEP BCI, an optimal value of each parameter can be attained. Experimental results of ten subjects showed that stimulus size of visual angle 3.8°, white, spatial proximity of visual angle 4.8° center to center apart, modulation sequence of length 63 bits and the lag of 4 bits between adjacent stimuli yield individually superior performance. These findings provide a basis for determining stimulus presentation of a high-performance c-VEP based BCI system.

## Introduction

Under normal circumstances, communication between the human brain and the external world is accomplished by peripheral nerves and muscles. However, people suffering from severe motor disability such as stem stroke, amyotrophic lateral sclerosis and cerebral palsy, lost autonomous control of their muscles, and thereby cannot communicate with the normal communication pathway. A brain-computer interface (BCI) translates intention into control commands of an external device and thus enables people to communicate without the involvement of peripheral nerves and muscles [1]. This feature makes BCIs popular in the field of neural engineering and clinical rehabilitation [2]. Electroencephalogram (EEG) recorded on scalp is widely used in BCI systems due to its non-invasiveness and ease to acquire. Although the performance of EEG based BCI systems has been improved considerably, they do not yet support widespread application in real-life environments.

Among various BCI paradigms, visual evoked potential (VEP) based BCIs have received increasing attention in recent decades [3–10]. VEPs are responses of the brain to visual stimuli and can be detected over the occipital lobe. VEPs evoked by stimulation of the central visual field are larger than those evoked by peripheral stimulation [11–12], and thereby can be controlled by a person’s gaze. According to specific modulation sequence of the stimulus, VEP based BCIs can be categorized as time modulated VEP (t-VEP) BCIs, frequency modulated VEP (f-VEP, i.e. SSVEP) BCIs and pseudorandom code modulated VEP (c-VEP) BCIs [3]. Among the three classes of BCIs, the latter two are the most potential BCIs that can achieve very high information transfer rate (ITR), which is the most widely used metric for a BCI system [1]. Unlike f-VEP BCIs, so far c-VEP BCIs have not yet been given a thorough study.

In a c-VEP BCI, a non-periodic binary code and its different time lags or multiple non-periodic binary codes of the same or different kinds are used to modulate different visual stimuli. When a person attends one of those stimuli, a c-VEP is evoked in the occipital lobe of the brain and can be detected with template matching. This idea was proposed by Sutter in 1984 [11] and was tested 8 years later on an amyotrophic lateral sclerosis (ALS) patient. Using intracranial electrodes for data acquisition, the subject was able to write 10 to 12 words/min [12]. Since then, the c-VEP BCI had seldom been investigated until recently. The study conducted by Bin et al. aroused great attention in BCI community by building a c-VEP BCI with a high ITR [13]. The average ITR achieved by their 32-target system was as high as 108 bits/min. Subsequently, four research groups reported their studies on c-VEP BCIs. Spuler et al made use of one-class support vector machines (OCSVM) for constructing templates and took advantage of error-related potentials for target recognition in order to improve the reliability and classification performance of c-VEP based BCIs [14–15]. An average ITR of 144 bit /min was achieved, which was the highest bit rate for a noninvasive BCI reported till then. Nezamfar et al. employed several M sequences to modulate visual stimuli in order to increase the number of command options [16]. Thielen et al. reported a novel c-VEP BCI paradigm that employed a generative framework to predict responses to broadband stimulation sequences. They designed a c-VEP BCI using modulated Gold codes to mark cells in a visual speller BCI [17]. Waytowich et al. also provided a novel c-VEP BCI paradigm that attempted to perform spatial decoupling of the targets and flashing stimuli via spatial separation and boundary positioning [18]. Experimental results showed classification accuracies for non-foveal condition comparable with those for direct-foveal condition for longer observation lengths.

Stimulus specificity is crucial for building a high-performance BCI. When a subject is presented with a visual stimulus, light information will arrive at the photoreceptors of the retina and then be transmitted to the visual cortex. When the brain is processing this information derived from vision, EEG can be captured in the occipital area of the scalp. EEG signals exhibit a number of features, and these features contain important information that can be translated into control signals. In the past decades, many research groups investigated the stimulus specificity of f-VEPs and f-VEP BCIs [19–25]. Based on their findings, it is established that altering stimulus properties such as the size and color of a fixated stimulus, or the number and separation of surrounding stimuli, will change the neuronal activities and resulting VEPs, and thus affect the classification performance of f-VEP BCIs. While stimulus specificity for f-VEP BCIs was well studied, that for c-VEP BCIs has not been explored yet. Since methods for target modulation and recognition of the two kinds of BCIs are different, their stimulus specificities are supposed to differ as well. c-VEP BCIs have excellent performance in terms of ITR, but many questions remain unanswered.

In a c-VEP BCI, stimulus specificity can at least be characterized by size, color and proximity of the stimuli, length of the modulation sequence and its lag between adjacent stimuli. Although the shape of stimuli has certain effect on target recognition, it is ignored in this study because square stimuli are in general used in c-VEP BCIs, especially for systems with a large number of stimulus targets. The former three parameters are common to f-VEP BCIs and c-VEP BCIs, whereas the latter two are specific to c-VEP BCIs. When these stimulus parameters are altered, EEG of the subject will be changed accordingly [19–25]. In this paper, a detailed investigation into the effect of stimulus specificity on target recognition is conducted so that the system performance can be improved in terms of classification accuracy.

## Materials and Methods

### 1. System configuration

The system consists of an EEG amplifier and a personal computer (PC) with a liquid crystal display (LCD) monitor. Stimulus presentation is operated in the PC and controlled by the system software developed by our team in Visual C++ environment. Either five or one stimulus is presented on the LCD monitor. Our c-VEP BCI requires a trigger signal in the EEG amplifier provided through the parallel port, which synchronizes the stimulus presentation and the EEG data recordings. Visual stimuli are presented on the LCD monitor with a refresh rate of 60 Hz and a resolution of 1920×1200 pixels. DirectX (Microsoft Inc.) is employed to ensure synchronization of the presented stimuli.

### 2. Experimental paradigms

The stimulus specificity of the c-VEP BCIs includes size, color and proximity of the stimuli, length and lag of the modulation sequence. Five experiments are designed to respectively explore the effect of each of these five parameters on the performance of c-VEP BCIs in terms of classification accuracy. These experiments were already conducted on separate days. While these parameters may interact with each other, they are analyzed separately for simplifying the analysis. A 63-bit binary M sequence and its time shifted versions are applied in all but the fourth experiment for the modulation of stimulus targets. Since square stimuli are most commonly used in VEP BCIs, they are used in all experiments in this study.

The stimulus parameters for each task are listed in Table 1. Depending on the experimental goal, the first two and the fourth tasks had only one stimulus target positioned at center of the screen with varying sizes, colors and sequence lengths respectively, whereas the third and fifth tasks had five stimulus targets with varying proximities and lags respectively. Note that the third task was a one-class classification problem although five targets were simultaneously presented on the screen. Because only one stimulus target was placed on the screen in experimental task 2 and 4, the largest stimulus size 8.9° in the first task was adopted to exclude the impact of stimulus sizes on target recognition; On the other hand, since five targets were placed on the monitor in the third and fifth tasks, the stimulus size of 3.8° was employed because it is optimal selection for multi-target systems according to the results of the first task.

**Table 1:** Five stimulus parameters used in the five experimental tasks.

Explored parameter	Stimulus parameters used in experiments					
	Size (visual angle)	Color	Proximity (visual angle)	Code length (bits)	Lag size (bits)	Number of classes
Size	0.67°, 1.7°, 2.8°,3.8°, 5.4°, 7.1°, 8.9°	white	-	63	-	1
Color	8.9°	Red, green, blue, yellow, white	-	63	-	1
Proximity	*3*.*8°*	white	3.8°, 4.8°, 5.8°, 6.8°	63	13	1
Code length	8.9°	white	-	15, 31, 63, 127	-	1
Lag size	*3*.*8°*	white	6.8°	63	2, 4, 6, 8,10	5

#### 2.1. Stimulus size

It is known that VEPs can be derived over the visual cortex during appropriate visual stimulation. When the size of the visual stimulus is altered, the VEP intensity will be changed accordingly [11, 26, 27]. The purpose of this experiment is to explore the effect of stimulus size on target recognition by placing a single, differently sized and white stimulus at the center of the screen without considering the interference from neighboring stimuli. The stimulus sizes measured in visual angle are 40′ (0.67°), 100′ (1.7°), 166′ (2.8°), 228′ (3.8°), 324′ (5.4°), 424′ (7.1°), and 536′ (8.9°), respectively. The choice of these sizes is related to the receptive field (RF) size of the human visual system [27]. The RF of a neuron is the spatial region where the firing of the neuron can be affected by the light stimulus [19]. The study by Kastner et al suggested that visual competition of multiple objects is in scale with RF size [20]. Thereby, the RF size must be considered in the study of the effect of stimulus size.

#### 2.2. Stimulus color

The color of visual stimuli is known to affect the elicited SSVEP in f-VEP BCIs, and a number of BCI studies have investigated which colors generate the strongest SSVEPs [21–25]. As for c-VEP BCIs, only one study investigated the effect of stimulus colors on BCI performance [28]. Since basic principles of these two systems are different, the best stimulus color for SSVEP BCIs may not mean best for c-VEP BCIs. The purpose of this experiment is to explore how the commonly used five colors, white, red, green, blue and yellow, influence target recognition. The experiment is conducted by placing a single, equivalently sized and differently colored stimulus at the center of the screen. The stimulus is flushed between a specific color and black with black background. A stimulus size of visual angle 8.9° is employed for the experimental task.

#### 2.3. Stimulus proximity

**Fig 1:**
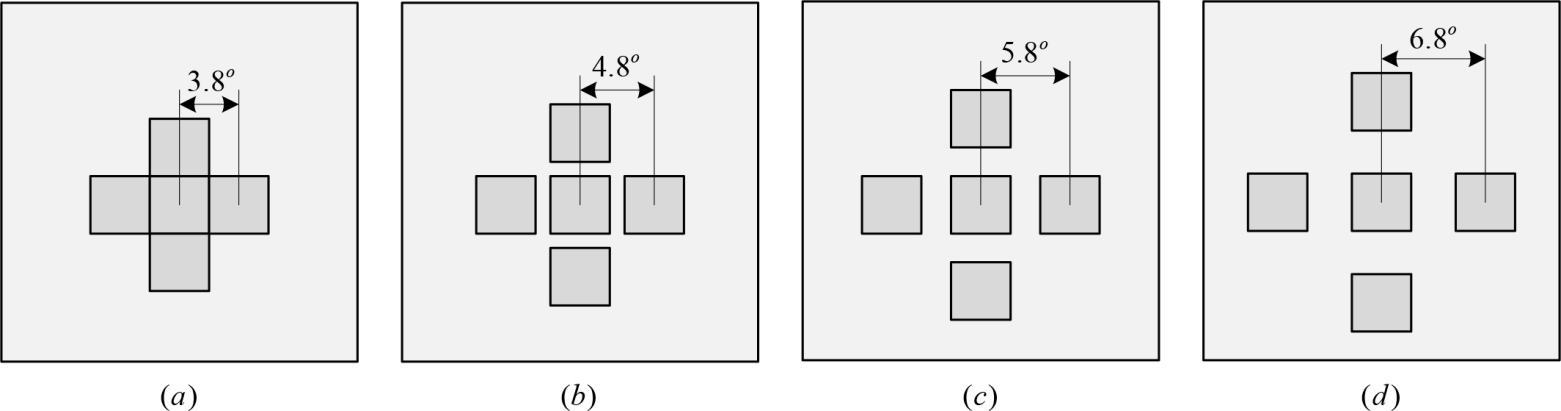
Five square stimuli with different proximities. The four competing stimuli have the same separation with the central stimulus in both horizontal and vertical directions. Stimuli in (a) are closely placed with visual angle 3.8°; Stimuli in (b), (c) and (d) are separately placed with visual angle 4.8°, 5.8° and 6.8°, respectively.

When a c-VEP BCI is employed to control a complex application with a large number of stimuli such as a mental speller, they can be closely placed according to principle of equivalent neighbors. However, when a c-VEP BCI is utilized to control a relatively simple application with a small number of stimuli such as wheelchair controlling, they can be positioned separately in order to eliminate mutual interference. The proximity of stimuli is the spatial distance between the stimulus being gazed at and the competing stimuli placed around it. The purpose of this experiment is to explore the effect of stimulus proximity on target recognition. A white stimulus with the size of 3.8° is placed at the center of the screen, and four white competing stimuli with the same size are placed equidistantly around the central one. The arrangement of these stimuli is shown in Fig 1. There are no competing stimuli placed in the diagonal positions, because their interference with the central stimulus is small and thus is negligible compared to competing stimuli in the horizontal and vertical positions. For convenience, the stimulus proximity we discussed here is defined as the center to center distance between two adjacent stimuli. Four different stimulus proximities, measured in visual angles, are 3.8°, 4.8°, 5.8° and 6.8° respectively.

#### 2.4. Length of the modulation sequences

In a c-VEP BCI system, we prefer short sequences to long ones because periodic stimulation modulated by a short sequence has a short stimulus cycle, which means that we need less time for target identification. However, too short modulation sequences may cause two problems: 1) it is probably difficult to discriminate between the template and its circular-shift counterparts, leading to low accuracy of target recognition; 2) short sequences have less circularly shifting sequences in the case of the same lag, which cannot modulate more stimuli. For example, a 31-bit binary M sequence can be shifted at most 14 times with 2-bit lag, and a set of 15 different modulation sequences can be obtained, which can be used to modulate at most 15 stimuli. The purpose of this experiment is to explore how length of a modulation sequence influences target recognition. The experiment is performed by placing a single, white stimulus with size of visual angle 8.9° at center of the screen, modulated respectively by M sequences of length 15 bits, 31 bits, 63 bits and 127 bits.

#### 2.5. Lag of the modulation sequence

The length and lag size of a modulation sequence are related to each other. For the same number of stimuli, longer modulation sequences have larger lags and vice versus. The purpose of this experiment is to explore how lag size of a modulation sequence affects target recognition. The experiment is performed by placing five stimuli on the screen according to the arrangement of Fig 1 with size of 3.8°, white color and proximity of 6.8°. All stimuli are modulated by a 63-bit binary M sequence and its time shifted versions. Since there are five stimuli in the experiment, the largest shift is up to 12 bits. The lag between adjacent stimuli investigated in the study is 2 bits, 4 bits, 6 bits, 8 bits and 10 bits respectively.

### 3. Target modulation and recognition

In an f-VEP BCI system, repetitive visual stimuli result in oscillatory EEG activity, which is closely correlated with the frequency of the observed stimulus, and thus frequency detection is used as the principle for classification. In our c-VEP BCI system, an M sequence and its time shifted versions are used for modulation of targets and template matching is adopted for recognition of the attended target.

#### 3.1. Target modulation

In the c-VEP BCI system, stimulus targets are modulated by an M sequence with length of 63 bits and its circular-shift counterparts in all but the fourth experiment, where the length of M sequences is respectively taken as 15 bits, 31 bits, 63 bits and 127 bits. The 63-bit M sequence used in our system is as follows

100000111000010010001101100101101011101111001100010101001111110

In our experiments, either one or five stimulus targets are employed for investigating stimulus specificity. Thereby, in the third experiment the time lag between two adjacent stimuli is 13 bits and that for each stimulus is decided by *τ*(*k*):

τ(k)=13×k,k=0,1,2,3,4 (1)

where *k* is the index of targets, and *k* = 0 corresponds to the central target. That is, the modulation sequences used for all targets were exactly the same except for the 13-frame time lag between two consecutive targets. In the fifth experiment, the time lag between two adjacent stimuli for five testing conditions (five subtasks) is 2 bits, 4 bits, 6 bits, 8 bits and 10 bits respectively.

#### 3.2. Canonical correlation analysis (CCA)

CCA is a multivariable correlation analysis method that finds underlying correlations between two multidimensional data sets. It creates a pair of linear combinations for two data sets such that the correlation between the two combinations is maximized [29]. Lin et al first applied CCA to off-line analysis of f-VEP BCIs [30] and the results demonstrated that the algorithm has much better robustness to noise than the commonly used fast Fourier transform (FFT) method. Bin et al employed CCA for online classification of stimulus targets and achieved a very high average ITR [31]. Subsequently, Bin et al adapted the algorithm for use in c-VEP BCIs [13]. Given two multidimensional variables *X* and *Y*, their respective linear combinations, referred to as canonical variants, can be denoted as *x* = *X*^*T*^*W*_*x*_ and *y* = *Y*^*T*^*W*_*y*_. CCA finds the two weight vectors *W*_*x*_ and *W*_*y*_ that maximize the correlation *ρ*_1_ between these two canonical variants, which is called canonical correlation. *W*_*x*_ and *W*_*y*_ are determined by solving the following optimization problem

$$\underset{W_{x},\;W_{y}}{\text{max}}\rho_{{}_{1}}(x,\;y)=\frac{E[x^{T}\;y]}{\sqrt{E[x^{T}\;x]E[{\text{y}}^{T}\;\text{y}]}}=\frac{E[W_{x}^{T}XY^{T}W_{y}]}{\sqrt{E[W_{x}^{T}XX^{T}W_{x}]E[W_{y}^{T}YY^{T}W_{y}]}}$$ (2)

where *W*_*x*_ and *W*_*y*_ are matrices and *ρ*_1_ is a one-dimensional vector. The first column of *W*_*x*_, denoted by *w*_*x*_, corresponding to the maximal value in *ρ*_1_, is used as the spatial filter.

#### 3.3. Target recognition

**Fig 2:**
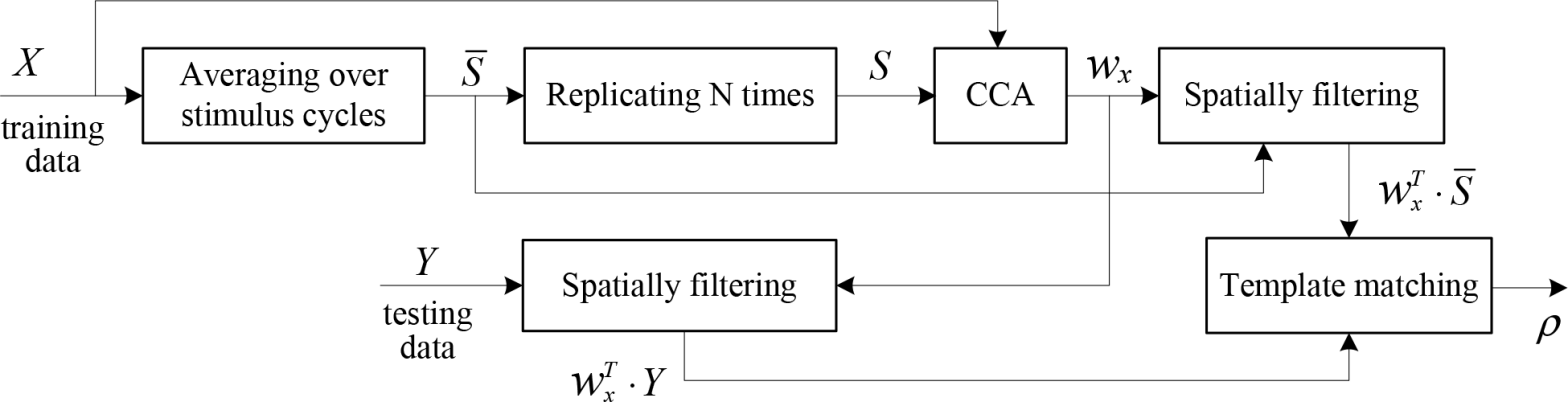
Flowchart of target recognition in the c-VEP BCI.

Target recognition includes two stages: a training stage and a testing stage. The former is utilized to create templates for all stimulus targets and design an optimal spatial filter for filtering both the reference template and currently recorded testing data, whereas the latter is employed to discriminate the stimulus target that the subject is gazing at. The flowchart of target recognition in the c-VEP BCI is illustrated in Fig 2.

In the training stage, the first step we need to do is to create a template for each stimulus target. A target *k*_0_ is specified as the reference target and the subject has to attend to the reference target for *N* stimulus cycles. The raw EEG data *X* are collected from multiple electrodes. They are segmented into single-cycle (i.e. single-trial) data *X*_*n*_, *n* = 1,2,⋯,*N* with dimension of *c* (channels)×*t* (time points). By averaging the EEG data from the *N* cycles, a multichannel template $\bar{S}$ (i.e. event-related potentials (ERP) signals) can be derived for the reference target

$$\bar{S}=\frac{1}{N}\sum_{\text{n}=1}^{N}X_{n}$$ (3)

The signal component *S* of the raw EEG data can be attained by replicating $\bar{S}$ for *N* times

$$S=[\bar{S},\;\bar{S},\;\cdots ,\;\bar{S}]$$ (4)

The dimension of both *X* and *S* is *c*×(*N*⋅*t*).

A one-dimensional template vector *T*_0_ for reference target *k*_0_ can be acquired by spatially filtering the multichannel template $\bar{S}$, i.e. $T_{0}=w_{x}^{T}\cdot \bar{S}$, where the spatial filter *w*_*x*_ is derived from CCA algorithm. Subsequently, templates for all other targets are generated by circularly shifting the one-dimensional reference template *T*_0_ according to the following equation:

$$T_{k}(t)=T_{0}(t-t_{s}(\tau_{k}-\tau_{0}))$$ (5)

where *τ*_*k*_ − *τ*_0_ computed by formula (1) indicates the time lag between target *k* and reference target *k*_0_, and *t*_*s*_ is the duration of one frame of the monitor. In our experiment, the refresh rate is 60Hz, thereby *t*_*s*_ = 1/60 = 0.0167 seconds. The length of templates *T*_*k*_ is *t* = 1050 corresponding to a stimulus cycle.

It is noted that like the third experiment, five templates are created in the former two and the fourth experiments although only one stimulus is presented. The other four templates are derived from the template of the presented stimulus by shifting it a certain number of bits. That is to say, one can imagine that the other four stimuli are also presented on the screen. In the former two experiments, the number of shifting bits for each imagined target is determined by formula (1); in the fourth experiment, the number of shifting bits between two neighboring targets is depended on the code length used in each subtask and is 3, 6, 13 and 25 bits for code length 15, 31, 63 and 127 bits respectively.

In the testing stage, the main task is to identify in real-time the target that the user is attending to. Template matching uses a similarity measure to match a signal to a template and is widely used in image processing [32–33]. It includes approaches such as normalized cross correlation (NCC), sum of absolute difference (SAD), sum of square difference (SSD), etc. In this study, the correlation method is adopted for target recognition. For a segment of currently recorded multichannel EEG data *x*_*c*_ with length of one stimulus cycle, it is first spatially filtered with the spatial filter *w*_*x*_, resulting in a one-dimensional data vector, $x=w_{x}^{T}\cdot x_{c}$. The correlation coefficients *ρ*_2_(*k*) between *x* and templates for all targets *T*_*k*_, *k* = 0,1,⋯,4, are calculated and the target with the largest coefficient is selected as the attended target

$$T_{a}=\underset{k}{\text{max}}\;\rho_{2}(k)$$ (6)

It is noted that in the former two and the fourth experiments, template matching is also conducted between one testing trial and each of the five templates and according to formula (6), the number of the template with maximal correlation coefficient is recognized as the presented target or one of the imagined targets. If the result is the presented target, it is decided as a correct recognition since only the presented stimulus is tested; if the result is one of the four imagined targets, it is decided as a wrong recognition.

### 4. Subjects and Data Acquisition

Ten healthy volunteers, six male and four female, named GHY, LXK, TDW, HY, DSF, DJ, YR, LYH, LJD and ZZ, were recruited to participate in the c-VEP BCI experiment. All subjects were asked to read and sign an informed consent form before participating in the study. Their average age was 23 years old with standard deviation 3 years. All subjects had normal or corrected to normal vision and had not suffered from any nervous diseases. During the experiment, each subject was seated in a comfortable armchair at 60 cm away from the computer monitor placed in a dimly lit, quiet room. The study was approved by the Human Research and Ethics Committee, Nanchang University.

The experiment consisted of five experimental tasks that are described in subsection Experimental paradigms. Each of the ten subjects completed the experiment (all five experimental tasks) on five days (sessions), one task per day. All sub-tasks in each experimental task, for example, every stimulus size in the first task, consisted of both training stage and testing stage. To avoid the effect of fatigue on the experiment, the subject was allowed to take a rest for 10 minutes between training stage and testing stage and for 5 minutes between sub-tasks in the testing stage. Each experimental task took 60~120 minutes, including the time for data acquisition, the time for experiment preparation (injection of electrode glue) and the time for relaxation. In the experiment, tasks were not counter-balanced, but sub-tasks were. In the second task, for example, the five colors (i.e. five sub-tasks) were tested by one subject according to the order of red-green-blue-yellow-white and by another subject according to the reversed order.

In the training stage, the sole target or central target was attended consecutively for *N* stimulus cycles and the recorded EEG data were used for constructing templates. The EEG recordings were segmented into single-cycle (i.e. single-trial) data, and a template was constructed by averaging the recorded EEG data across stimulus cycles and then spatially filtering the averaged data. The number of stimulus cycles was determined experimentally as 200 that could ensure good templates for target recognition; In the testing stage, for the former four experimental tasks, the sole target (in task 1,2 and 4) or the central target (in task 3) was attended consecutively for 40 stimulus cycles for each sub-task; for the fifth experimental task, each of the five targets was attended consecutively for 40 cycles for each of the five sub-tasks (i.e. five lags). The order of the target to be attended was random. The EEG recordings were also segmented into single-cycle (i.e. single-trial) data, which the target recognition was based on. The total number of testing trials conducted for each of the former four tasks was 40 multiplied by the number of sub-tasks, while that conducted for the fifth task was 40 times the number of sub-tasks times the number of targets.

**Fig 3:**
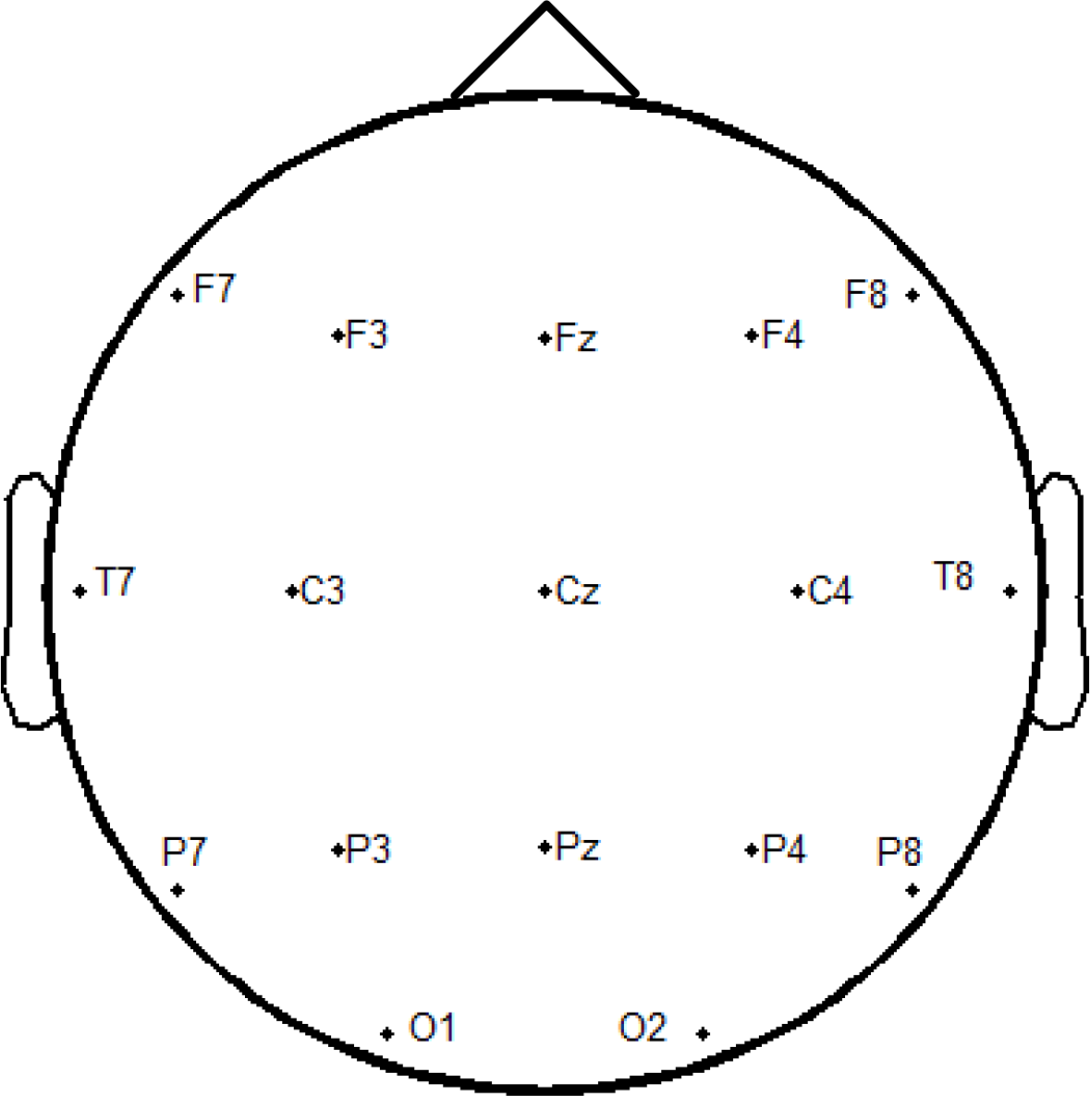
The topographic map of sixteen signal electrodes and the ground electrode (Fz). The seven electrodes over the occipital region (P7, P3, Pz, P4, P8, O1, O2) were selected for data recordings in the experiment.

The EEG signals were recorded with a 16-channel Ag/AgCl electrode cap and digitized at 1000 Hz with a MiPower amplifier made in the Institute of Neural Engineering, Tsinghua University, China. As shown in Fig 3, seven electrodes over the occipital region (P7, P3, Pz, P4, P8, O1, O2) in line with international 10/20 system were selected for data recordings in the experiment [34]. The ground electrode was positioned at Fz and the reference electrode was placed at the mastoid of left ear. The EEG recordings were preprocessed by data intercepting of single trials, data detrending and band-pass filtering between 2Hz and 30Hz using an infinite impulse response (IIR) of order eight. The preprocessed EEG data were employed for offline analysis of stimulus specificity of the c-VEP BCI.

## Results and Analysis

The evaluation criterion of the c-VEP BCI performance was classification accuracy, which was defined as the number of correctly recognized trials divided by the total number of trials conducted for one experimental subtask.

### 1. Stimulus size

**Fig 4:**
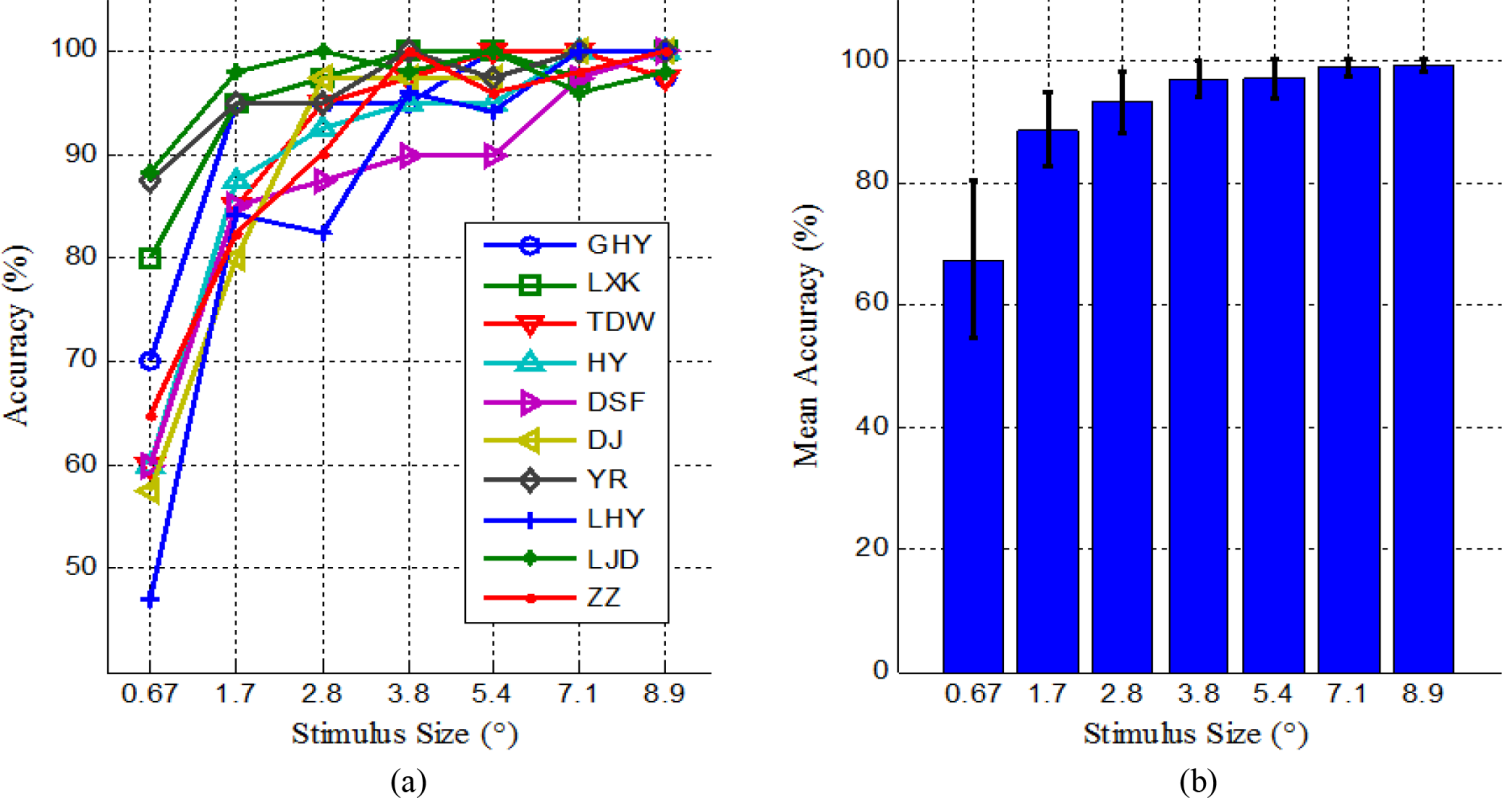
(a) The classification accuracy of each subject as a function of stimulus size; (b) Average classification accuracy across subjects for each stimulus size. The error bars represent standard deviation.

The classification accuracy of each subject as a function of stimulus size is shown in Fig 4(A). It is seen that although there is inter-subject variability, most accuracy curves first increase monotonically with stimulus size and then vary in a small range. A close look reveals that for all subjects, a rapid rise in accuracy occurs when stimulus size is small. In the size of visual angle 1.7°, the accuracies of four subjects already reached to a very high level of above 90%; In a medium size of 3.8°, only one subject yielded an accuracy below 90%; When the size changed to 7.1°, the performance of all subjects is satisfactory with accuracy above 95%. The average accuracy rate across subjects for each stimulus size is shown in Fig 4(B). The error bars represent standard deviation. It can be observed that there is a clearly upward trend in the accuracy rate as the size of the stimulus increases, and the standard deviation decreases accordingly.

Wilcoxon signed-rank test for a statistical analysis of median difference significances was applied to investigate the effect of stimulus specificity on classification accuracy, because all the accuracy rates were not normally distributed. The paired test at 95% confidence level was conducted to examine statistically significant difference between any two of the seven sizes. The results indicated that the size of 0.67° was significantly worse than all six larger sizes (p = 0.005), the size of 1.7° was significantly worse than all five larger sizes (*p* = 0.025, 0.011, 0.005, 0.007 and 0.008 respectively), and the size of 2.8° was significantly worse than all four larger sizes (*p* = 0.016, 0.011, 0.015 and 0.007 respectively). There were no significant differences between the size of 3.8° and the three larger sizes (*p* = 0.916, 0.065 and 0.052 respectively), between the size of 5.4° and all two larger sizes (*p* = 0.159 and *p* = 0.083 respectively), and between the size of 7.1° and the size 8.9° (*p* = 0.746). As a result, the stimulus size of 3.8° can be used for stimulus design in a c-VEP BCI system.

### 2. Stimulus color

**Fig 5:**
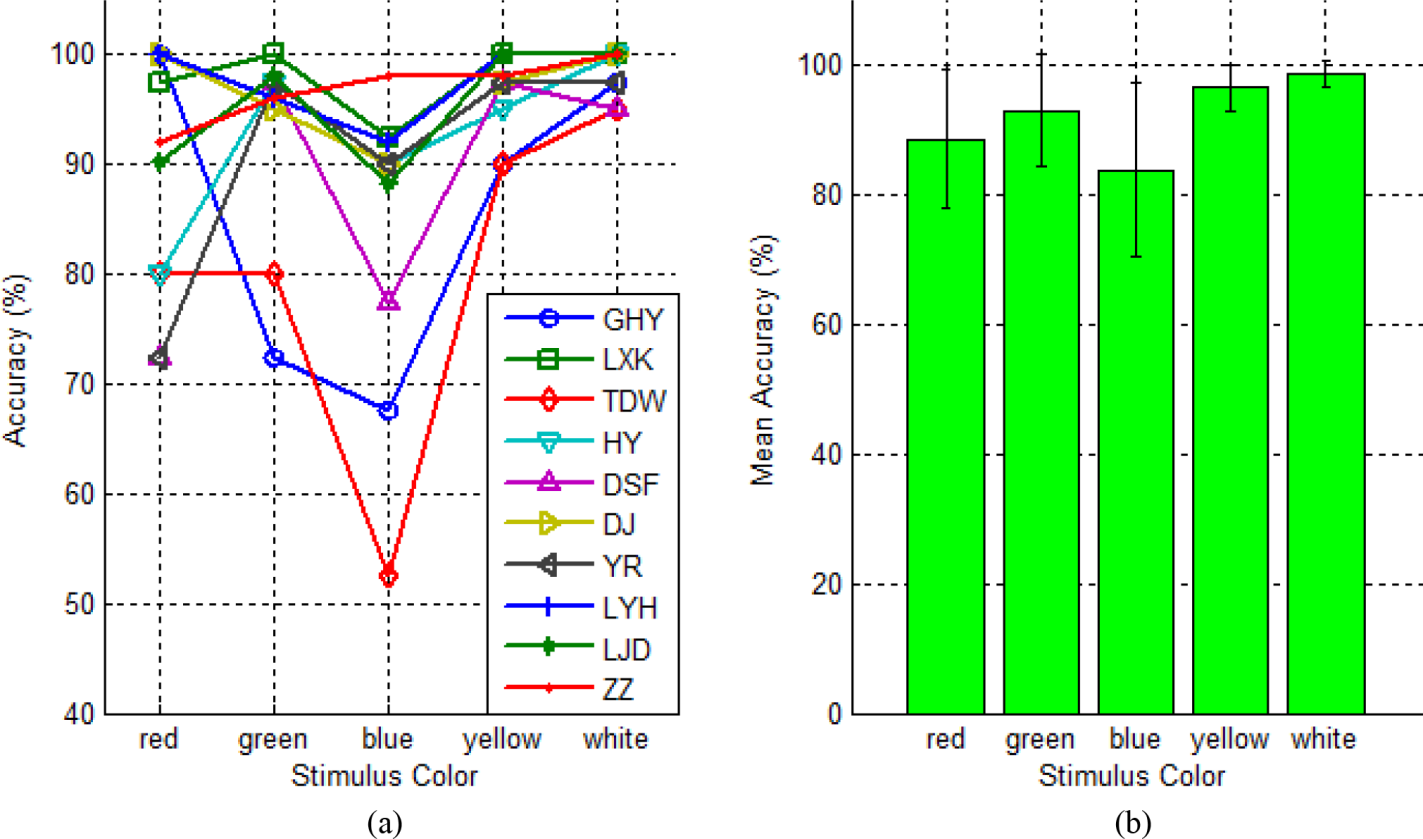
(a) The relationship between classification accuracy and stimulus colors for each subject.; (b) Average classification accuracy across subjects for each stimulus color. The error bars represent the standard deviation.

Fig 5(A) illustrates the relationship between classification accuracy and stimulus colors for each subject. It is observed from the subplot that although response of each subject to stimuli of the five colors is different, all subjects but ZZ yielded the lowest classification accuracies at blue color. Three subjects (GHY, DJ and LYH) attained the highest accuracy at red colors, three subjects (LKX, DSF and YR) achieved the highest accuracy at green color, no subjects yielded the highest accuracy at blue color, five subjects (LXK, DSF, YR, LYH and LJD) obtained the highest accuracy at yellow color and seven subjects (LXK, HY, DJ, YR, LYH, LJD and ZZ) yielded the highest accuracy at white color. Fig 5(B) illustrates the average classification accuracy across subjects for each stimulus color. The error bars represent standard deviation. It is observed from the subplot that the accuracy rate yielded by white is the highest, followed by yellow, green and red, and blue achieved the lowest accuracy rate. The two best colors yielded accuracy rates higher than 95%. On the other hand, the standard deviations of accuracy for the five colors were inversely proportional to their accuracy rates.

Paired Wilcoxon signed-rank tests at 95% confidence level were carried out to examine statistically significant differences between any two of the five colors. The results revealed that white was significantly better than red, green and blue with p = 0.021, 0.003 and 0.005 respectively, and yellow and green were significantly better than blue with p = 0.007 and p = 0.007 respectively. Since in terms of classification accuracy, there were no statistically significant differences between white and yellow (p = 0.092), the two colors can be individually used as the stimulus color in a c-VEP BCI system.

### 3. Stimulus proximity

**Fig 6:**
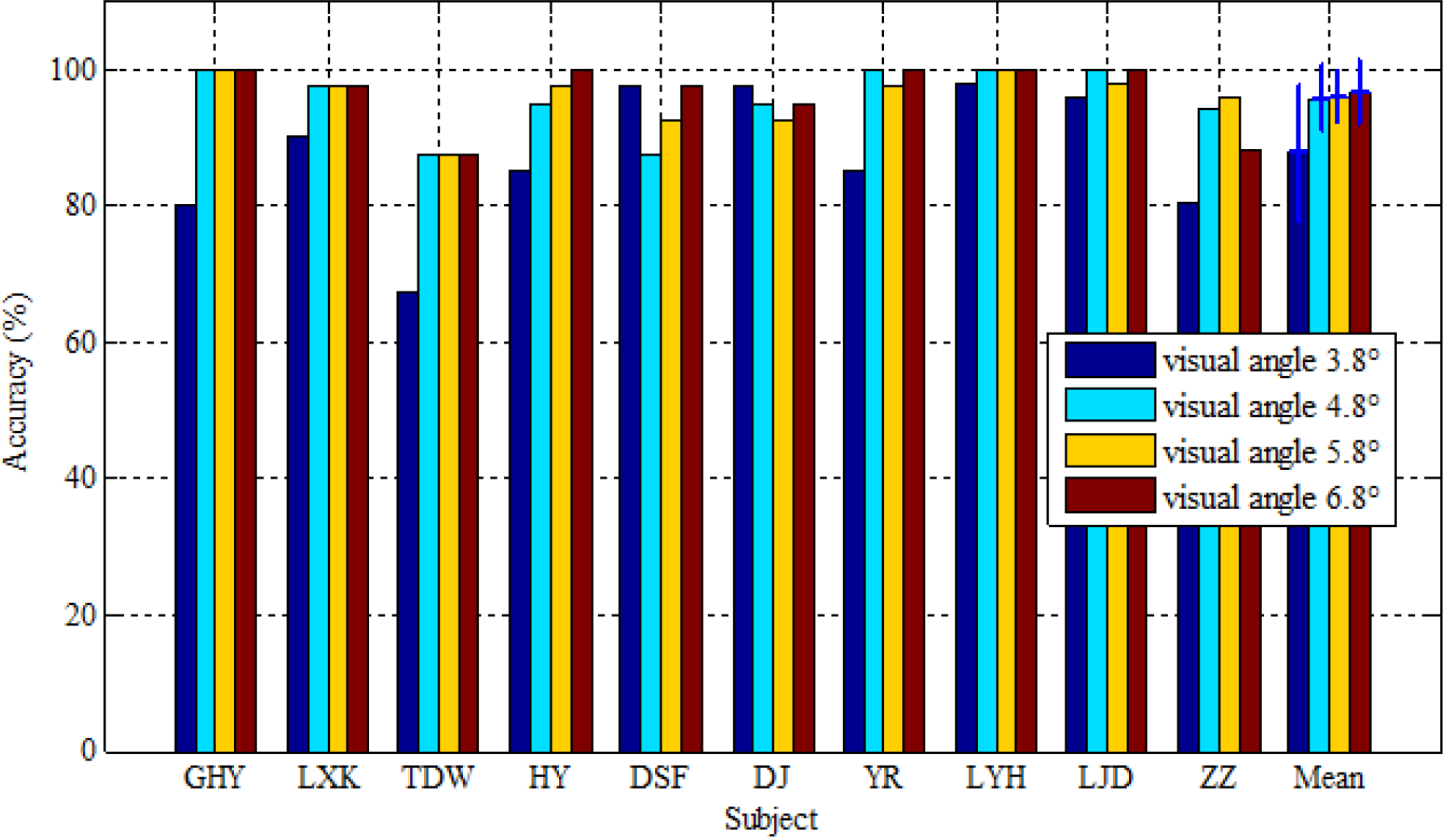
Classification accuracy of each subject and their average at four different proximities measured by center to center distance and denoted by visual angles 3.8°, 4.8°, 5.8° and 6.8°. The error bars represent standard deviation.

The experimental task was to investigate the effect of mutual interference of adjacent stimuli on the classification performance of c-VEP BCIs when the five stimuli were placed in different separations. The sizes of these stimuli must be selected properly to guarantee high performance in terms of classification accuracy. The sizes and colors of these five stimuli were set at a visual angle of 3.8° and white respectively in the experimental task. Fig 6 depicts the classification accuracy of each subject and their average at four different proximities denoted by visual angles 3.8°, 4.8°, 5.8° and 6.8°. The error bars stand for standard deviation. Note that the accuracy was measured by the central stimulus. From this figure it is observed that at proximity 3.8°, accuracy is the lowest for all subjects except for subjects DSF and DJ. When the proximity increased to 4.8°, accuracy was considerably improved for most subjects due to sharp fall of interference. Greater separation generated small or no improvement in accuracy because of little interference with central stimulus.

Paired Wilcoxon signed-rank tests at 95% confidence level were conducted between any two of the four proximities. The results indicated that in terms of classification accuracy, all three greater proximities were significantly better than the proximity of visual angle 3.8° with *p* values equaling 0.041, 0.036 and 0.015 respectively. There were no significant differences among the latter three stimulus proximities in terms of classification accuracy. It is naturally inferred from the results that tightly arranged stimuli inevitably cause mutual interference and stimulus targets in our c-VEP BCI should be at least 4.8° center to center apart when their sizes are fixed at 3.8° in order to achieve good accuracy.

### 4. Length of stimulus code

**Fig 7:**
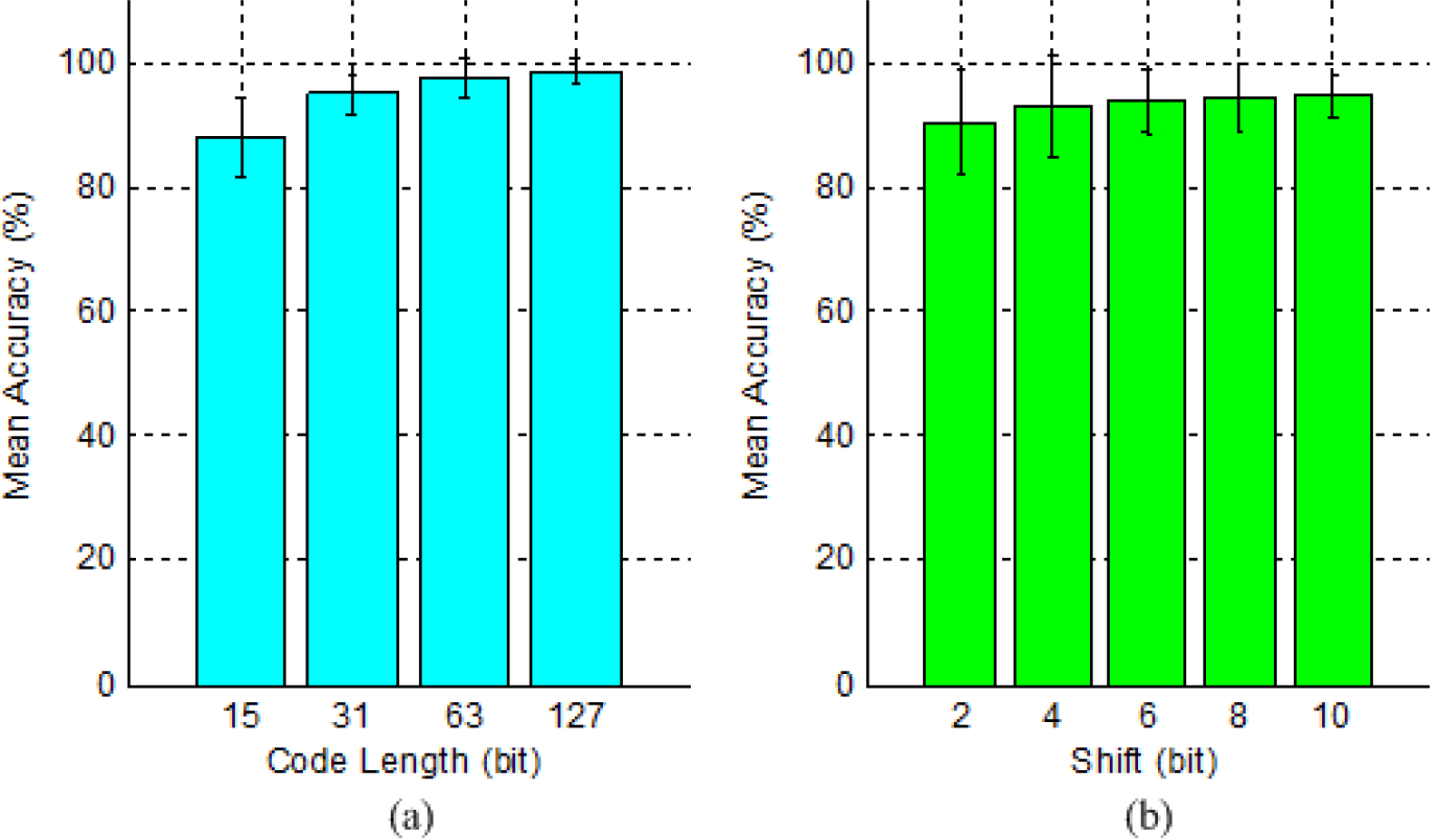
Average classification accuracy across subjects yielded by different lengths of M sequences (i.e. code lengths) (a) and different versions of shifts of the 63-bit M sequence between two adjacent targets (b).The error bars denote standard deviation.

In our multi-target c-VEP BCI system, targets are modulated by a fixed length of M sequence and its circular shifting versions. Thus, the length of stimulus code and the size of shifting (i.e. lag) are two interrelated parameters that have important influence on target recognition. The experimental task of code length is to determine the shortest code length that ensures high classification performance for a single target. The average classification accuracy across subjects yielded by different lengths of M sequences is shown in Fig 7(A). Clearly, the average accuracy increases constantly with code length, but the increasing speed slows down with code length. When code length was 15 bits, the average accuracy was only 87.8%; when code length increased from 15 bits to 31bits, 63 bits and 127 bits respectively, the average accuracy first jumped to 94.9%, and then increased slowly to 97.4% and 98.5%.

Paired Wilcoxon signed-rank tests at 95% confidence level were conducted between any two of the four code lengths. The results revealed that in terms of classification accuracy, the code length of 15 bits was significantly worse than all three greater code lengths with *p* values equaling 0.011, 0.005 and 0.005 respectively, and the code length of 31 bits was significantly worse than all two greater code lengths with *p* value equaling 0.008 and 0.026 respectively. There was no statistically significant difference between code length 63 bits and 127 bits with *p* value equaling 0.285. Thus, the code length of 63 bits is the good choice for building a multi-target c-VEP BCI system.

### 5. Shift of stimulus code

The experimental task of shifting size is to determine the smallest lag of the stimulus code between two adjacent targets that does not significantly affect target identification. The size, color and proximity of these targets were respectively set as visual angle of 3.8°, white and visual angle of 6.8°. The average classification accuracy across subjects yielded by the 63-bit M sequence and its different versions of time shifting is shown in Fig 7(B). Note that this is a five-class classification problem and thus the accuracy was averaged with the accuracies of all five targets. It is observed that the average accuracy improved constantly with the size of shifting, but the improving speed became increasingly slow.

Paired Wilcoxon signed-rank tests at 95% confidence level were conducted between any two of the five shifting sizes. The results revealed that in terms of classification accuracy, the shifting size of 2 bits was significantly worse than all greater shifting sizes with *p* values equaling 0.011, 0.033, 0.012 and 0.036 respectively. There were no statistically significant differences among the latter four shifting sizes. Although the average accuracy reached 90.4% with the shifting size of 2 bits for the five-target c-VEP BCI, it might decrease for systems with more targets. Since no significant differences existed among the latter four lag sizes, at least 4-bit shifting is needed in a multi-target c-VEP BCI system in order to ensure highest classification performance.

## Discussion

Different modulation methods require different methods for target recognition that result in different system performance. The modulation signals of f-VEP BCIs are periodic square-wave signals at specific frequencies higher than 6 Hz, and the frequency spectra of elicited f-VEP signals exhibit sharp spectral peak at the stimulus frequency and its harmonics. The modulation signals of c-VEP BCIs are a binary sequence and its different time lags or multiple unique binary sequences, and the frequency spectra of elicited VEP signals are of wide band characteristic without sharp spectral peaks at any frequencies. Accordingly, the methods for target recognition of the two kinds of BCIs are totally different. Target recognition in f-VEP BCIs is conducted by frequency and/or phase detection, whereas that in c-VEP BCIs is usually done by template matching. Their differences in modulation signals and target recognition inevitably lead to difference in optimal stimulus parameters.

A robust optimization of parameters should consider the possible interactions among them. In the study, each of these five parameters was varied separately, which related to the assumption that there were not any interactions. However, testing this hypothesis directly would require a large amount of repetitions of the experiment with different combinations of parameters. In such setup, a complete search of the solution space would require up to dozens of days of signal acquisition for each subject, which obviously exceeds the possibilities and aims of this research.

For building a c-VEP BCI, the first thing one must consider is to determine the size of stimuli. The results shown in Fig 4 revealed that in the range of visual angle 0.67° to 8.9°, the larger stimulus was more effective as for target recognition. This is in line with previous studies on f-VEP BCIs conducted by Duszyk [25], Busch [26] and Ng [27]. There is no established theory to explain the phenomenon, but Busch attributed it to the assumption that larger area in the visual cortices is activated by larger stimuli. However, when stimulus dimension was larger than 3.8°, its influence on accuracy became increasingly insignificant. As a result, if other factors such as visual fatigue are not taken into account, the dimension of stimuli is at least visual angle of 3.8° in order to ensure high classification performance. This is in discordance with Ng’s result derived from f-VEP BCIs, in which it is necessary for the stimulus size to subtend at least visual angle 2°.

Stimulus color is the next parameter that has significant effect on the performance of BCI systems. Five different colors commonly used in BCI systems were tested with black background. The results illustrated in Fig 5 demonstrate that for c-VEP BCIs, the two mixed colors, white and yellow performed better than three basic colors. This could be explained from the point of view of anatomy. The perception of light for human eye is achieved through the retina, which contains two kinds of cells: rod and cone. The function of rod cells is to sense the luminance of outside world, whereas the function of cone cells is to distinguish different colors. The retina contains three different kinds of cone cells, red, green and blue. Hence, human eyes have different response to light stimulus of different colors. Since white consists of three basic colors, it can simultaneously stimulate these three kinds of cone cells and as a result, strongest c-VEP signal is acquired with white light. Similarly, yellow consists of red and green and thus the second strongest c-VEP signal is attained with yellow light. In contrary to previous study on f-VEP BCIs [25], the study exhibited that green was a more effective stimulus color than red, although the difference in accuracy was not significant. This might be caused due to their difference in principle of the two kinds of BCIs. Recently, Amanika et al. presented a novel c-VEP BCI paradigm using four green-blue chromatic flashing stimuli in order to minimize the danger of photosensitive epilepsy [28]. They compared the accuracy of the green-blue stimuli with that of conventional white-black stimuli, and the result indicated that the former was not a patch on the latter, whether the stimuli were in low frequency or in high frequency. The two c-VEP BCI studies conducted by Bin et al [13] and Spuler et al [15] achieved high classification accuracy and ITR using white-black stimuli, partially proving the validity of our results, i.e. white is the most effective stimulus color in a c-VEP BCI.

Proximity among stimuli is actually related to size of stimuli. In this study, the size of stimuli was fixed to 3.8° of visual angle and four different proximities 3.8°, 4.8°, 5.8° and 6.8°, measured by center to center distance between two stimuli, were tested. The results revealed that accuracy yielded by tightly placed stimuli (i.e. proximity of visual angle 3.8°) were significantly lower than that achieved by separately placed stimuli and thus stimuli in a c-VEP BCI should be kept at least 4.8° apart to avoid interference from neighboring stimuli. While the principle of equivalent neighbors and template matching can alleviate the impact of interference on target recognition [12–13], it seems difficult to fully eliminate this kind of interference. In previous studies on stimulus specificity based on f-VEP BCIs, two authors drew two opposite conclusions. Ng [27] thought the influence of inter-stimulus distance on SSVEP amplitude was significant, whereas Duszyk believed it was not [25]. Duszyk attributed the reason to the methodological difference between the two studies: one directly measured the amplitude of SSVEP response, and the other calculated classification accuracy. Another reason might be the size of stimuli used in the two proximity experiments. The larger the stimuli were, the smaller the interference of neighboring stimuli was, because human eyes are much more sensitive to light entering from center of the pupil than from off-center rays [35–36], and the evoked response decreases as a Gaussian function of stimulus width from the fovea to about 5° of visual angle [37]. Considering more targets can be arranged on the screen, the stimulus was set as a medium size in our experiment, and thus closely placed competing stimuli yielded interference on the central one.

The last two stimulus parameters tested in this study are the code length and shifting size. They interact with each other and have important impact on system performance of c-VEP BCIs. ITR, as a standard metric of BCI systems, is determined by the number of choices/stimuli, the accuracy of target detection and the average time taken for a selection/detection [1]. To enhance ITR, the number of choices should be large, the accuracy of target detection should be high and the average time for a selection should be small. As shown in Fig 7(A), the detection accuracy increased with code length, but the increase in accuracy was insignificant when code length reached 63 bits. Since target detection was done using a whole stimulus cycle, the detection time also increased with code length. Thereby, a 63-bit code length is suitable for c-VEP BCIs to compromise between detection accuracy and detection time. As shown in Fig 7(B), the detection accuracy increased with shifting size when code length was fixed to 63 bits, but the increase in accuracy was insignificant when the shifting size reached 4 bits. Because large shifting size resulted in a fewer number of circularly-shifting codes, the available number of stimuli decreased with shifting size as well. Thereby, a shifting size of 4 bits is appropriate to compromise between detection accuracy and the number of stimuli.

## Conclusions

A visual stimulator is an essential component of a VEP BCI system, and its stimulus specificity has an important impact on the overall system performance. In this paper, we characterized the stimulus specificity of c-VEP BCIs with five main parameters, i.e. the size, color, and proximity of the stimuli, length and lag of the stimulus sequence. To build a c-VEP BCI, the first thing one must take into account is to determine the these stimulus parameters. Based on the assumption of lack of the interactions among these five stimulus parameters, we carried out a detailed study on stimulus specificity of c-VEP BCIs by exploring how it influences the classification accuracy.

Each of these five stimulus parameters provided optimal performance based on other four parameters. In a single-target system or a system with a few targets, white color should be the first choice, the dimension of stimuli, the distance between stimuli and the lag of M sequence should be selected as large as possible, and the M sequence should be selected as long as possible. In a multi-target system, stimulus size of 3.8°, white, spatial proximity of 4.8° center to center apart, M sequence of length 63 bits and the lag of 4 bits between adjacent stimuli yield individually superior performance. These results provide a good advice to the ones who want to construct a c-VEP BCI system.

## Supporting Information

S1 File The results of target recognition in the five experimental tasks: size, color, proximity, code length and code shift of the stimuli. (XLS)
